# Combination of a thioxodihydroquinazolinone with cisplatin eliminates ovarian cancer stem cell-like cells (CSC-LCs) and shows preclinical potential

**DOI:** 10.18632/oncotarget.23679

**Published:** 2017-12-26

**Authors:** Jing Ma, Joseph Salamoun, Peter Wipf, Robert Edwards, Bennett Van Houten, Wei Qian

**Affiliations:** ^1^ Department of Pharmacology and Chemical Biology, University of Pittsburgh, and UPMC Hillman Cancer Center, Pittsburgh, PA 15213, USA; ^2^ Department of Respiratory and Critical Care Medicine, Tongji Hospital, Tongji Medical College of HuaZhong University of Science and Technology, Wuhan 430030, China; ^3^ Department of Chemistry, University of Pittsburgh, Pittsburgh, PA 15260, USA; ^4^ Accelerated Chemical Discovery Center, University of Pittsburgh, Pittsburgh, PA 15260, USA; ^5^ Department of Obstetrics and Gynecology, University of Pittsburgh Medical Center, Pittsburgh, PA 15213, USA

**Keywords:** thioxodihydroquinazolinone small molecule, cisplatin resistance, ovarian cancer stem cell-like cells, cancer spheroids, apoptosis

## Abstract

Cancer stem cell-like cells (CSC-LCs) contribute to drug resistance and recurrence of ovarian cancer. Strategies that can eradicate CSC-LCs are expected to substantially improve the outcome of ovarian cancer treatment. We have previously identified a class of thioxodihydroquinazolinone small molecules, which have strong synergistic antitumor activity with platinum drugs, the standard chemotherapeutic agents for ovarian cancer treatment. In the current study, using the activity of aldehyde dehydrogenase (ALDH) as a marker of CSC-LCs, we demonstrated that the combination of thioxodihydroquinazolinone compound 19 with cisplatin is able to diminish ALDH-high CSC-LC populations in both platinum-resistant ovarian cancer cell lines and primary ovarian cancer cells from metastatic ascites of a cisplatin-resistant patient. Compound 19 enhanced the accumulation of intracellular cisplatin in ALDH-high ovarian CSC-LCs. The combination of compound 19 with cisplatin was also able to reduce the sphere-forming capability of cisplatin-resistant ovarian cancer cells. Using a spheroid-based *in vitro* metastasis model of ovarian cancer, we demonstrated that the co-administration of compound 19 with cisplatin prevents ovarian cancer spheroid cells from attaching to substratum and spreading. In a cisplatin-resistant *in vivo* intraperitoneal xenograft mouse model, the combination of compound 19 with cisplatin significantly reduced tumor burden, as compared to cisplatin alone. Taken together, our study demonstrated that thioxodihydroquinazolinones represent a new class of agents that in combination with cisplatin are capable of eliminating CSC-LCs in ovarian cancer. Further development of thioxodihydroquinazolinone small molecules may yield a more effective treatment for cisplatin-resistant metastatic ovarian cancer.

## INTRODUCTION

Ovarian cancer is the leading cause of death among gynecological malignancies. Surgical debulking followed by platinum- and taxane-based chemotherapy is the current treatment standard for advanced ovarian cancer. Despite the high response rate to initial chemotherapy including complete responses, intrinsic and acquired drug resistance result in disease recurrence. Platinum-resistant ovarian cancer is uniformly fatal [1]. Drug resistance remains as a major challenge to the treatment of ovarian cancer. Elucidating the underlying mechanism of drug resistance and developing effective strategies to overcome resistance are high priorities in both investigational and clinical oncology of ovarian cancer.

Among the many factors that contribute to platinum drug resistance, ovarian cancer stem cell-like cells (CSC-LCs), or tumor initiating cells, which have the ability to repopulate the entire tumor after therapeutic treatments, are recognized as one of the critical underlying mechanisms and a potential target for treating ovarian cancer [2, 3]. Ovarian CSC-LCs are defined by the expression of several molecular markers, such as aldehyde dehydrogenase isoform 1 (ALDH1), CD133, and CD117 [4–6]. Characteristics of ovarian CSC-LCs, such as the population size of CSC-LCs [7] and the expression of CD44 [8], have been proposed to serve as predictive markers for disease progression and to aid in the treatment selection. Since chemotherapy-resistant ovarian cancer cells are enriched in CSC-LCs [9], CSC-LCs are believed to contribute to the aggressive behavior and recurrence of ovarian cancer [10–12]. The resistant phenotype of CSC-LCs can be attributed to several factors, including reduced intracellular drug accumulation, enhanced DNA repair, and quiescent state of CSC-LCs [13]. Strategies targeting CSC-LCs have also been explored in order to reverse drug resistance, such as targeting surface stem cell markers, inhibiting drug efflux transporters, modulating self-renewal and differentiation pathways, and inducing apoptosis [14]. Particularly in ovarian CSC-LCs, inhibition of Notch activity by γ-secretase inhibitors (GSI) [15], inhibition of Aurora A kinase [16], or targeting epigenetic processes by DNA methyltransferase inhibitors [9] have been shown to assist in overcoming platinum resistance.

**Figure 1:**
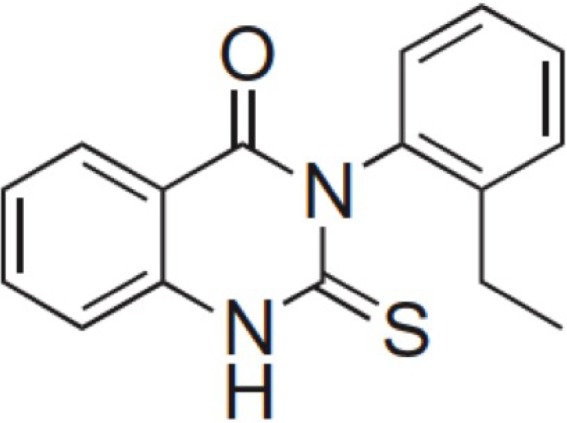
Structure of thioxodihydroquinazolinone compound 19

Our previous work on small molecule screening and functional studies led to the discovery of a series of thioxodihydroquinazolinones, which elicit a synergistic effect with platinum anticancer drugs to induce apoptotic cell death in platinum- and multidrug- resistant cancer cells, including ovarian cancer cells [17, 18]. To further explore the therapeutic potential and elucidate the mechanism underlying the anticancer activities of those thioxodihydroquinazolinones, we hypothesized that these small molecules are able to effectively eradicate CSC-LCs, thereby overcoming drug resistance and preventing tumor recurrence. We have shown previously that compound 19 (Figure 1) is one of the most potent thioxodihydroquinazolinone analogs when combined with platinum drugs in inducing apoptotic cell death in platinum-resistant A2780cis human ovarian cancer cells [17]. In the current study, using compound 19 as a lead compound we demonstrated that the combination with cisplatin exhibits a strong inhibitory effect in ovarian CSC-LCs.

## RESULTS

### The combination of compound 19 with cisplatin effectively diminishes the cell sub population of ovarian cancer stem cell-like cells (CSC-LCs)

**Figure 2:**
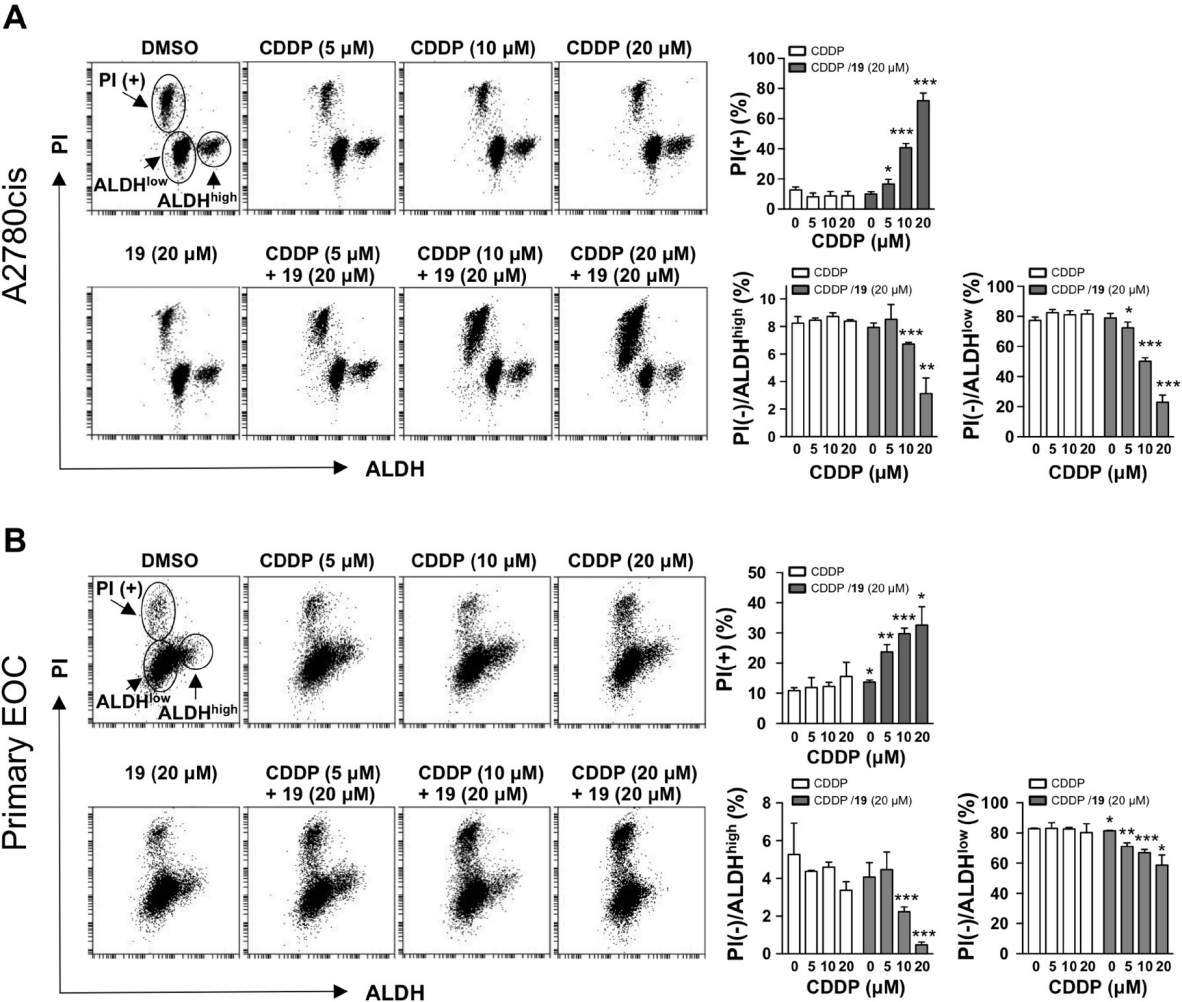
The combination of compound 19 with cisplatin diminishes ovarian cancer ALDH^high^ stem cell-like cells (CSC-LCs) population Cisplatin-resistant A2780cis ovarian cancer cells (**A**) and primary epithelial ovarian cancer (EOC) cells from ascites of a cisplatin-resistant ovarian cancer patient (**B**) were treated as indicated for 24 h. ALDH activity was assessed using an ALDEFLUOR kit followed by flow cytometry. Propidium iodide (PI) was used to quantify cell survival. The percentages of live cells (PI negative) with high or low ALDH activity, and the percentages of dead cells (PI positive) were quantified. Data represent mean ± SD from three independent experiments. Student’s *t*-test was performed for comparisons between groups treated with the combination of cisplatin (CDDP) with compound 19 and groups treated with cisplatin alone at the same concentration. ^*^P < 0.05, ^**^P < 0.01, ^***^P < 0.001.

The activity of ALDH has been exploited as a marker defining multiple types of CSC-LCs, including ovary, breast, and brain CSC-LCs [9, 19, 20]. It has also been shown that after platinum treatment, residual ovarian cancer xenografts are enriched with cells expressing high ALDH activity [9]. Ovarian cancer cells with high ALDH activity also showed increased chemoresistance [20]. We therefore employed an ALDH activity assay to determine the stem cell-like populations in cisplatin-resistant A2780cis ovarian cancer cells, and CSC-LCs were identified as cell populations expressing high ALDH activity. We then used propidium iodide (PI), which is not membrane permeant to viable cells but stains DNA in dead cells, to monitor cell death induced by drugs alone or in combination in both CSC-LCs (ALDH^high^) and non-CSC-LCs (ALDH^low^). As shown in Figure 2A, treatment with cisplatin alone at up to 20 μM for 24 h did not induce a significant cell death in A2780cis cells as indicated by a lack of increase in the PI-positive (PI+) cell population, which is consistent with the platinum-resistant phenotype of A2780cis cells. Cisplatin alone and compound 19 (20 μM) alone also did not reduce the survival of CSC-LCs, as indicated by the levels of PI(−)/ALDH^high^ population. In contrast, the co-treatment of compound 19 and cisplatin not only significantly reduced overall cell viability (as indicated by an increase in the PI(+) population), but also significantly reduced the PI(−)/ALDH^high^ CSC-LCs population in a dose-dependent manner (a 63% reduction was observed after the treatment with the combination of 20 μM of both agents as compared to the treatment with 20 μM cisplatin alone). In addition, the viability of non-CSC-LCs as indicated by the PI(−)/ALDH^low^ population was also reduced by the combination treatment. We further confirmed the effect of the combination of compound 19 and cisplatin on the ALDH^high^ CSC-LCs population by utilizing primary epithelial ovarian cancer (EOC) cells that we isolated from the ascites fluids of a cisplatin-resistant ovarian cancer patient (Figure 2B). Similar to Figure 2A, the combination significantly reduced the ALDH^high^ CSC-LCs population as compared to the treatment with cisplatin or compound 19 alone (Figure 2B). This result indicated that compound 19 represents a new class of small molecules that, when combined with cisplatin, are able to deplete the drug-resistant ALDH^high^ CSC-LCs population, as well as the ALDH^low^ non-CSC-LCs population. Since targeting the CSC-LCs population alone might be less efficient in total tumor eradication [15], the ability of the combination of compound 19 with cisplatin in inducing cell death in both CSC-LCs and non-CSC-LCs may represent a therapeutic advantage.

### Compound 19 enhances accumulation of intracellular cisplatin in ovarian CSC-LCs

**Figure 3:**
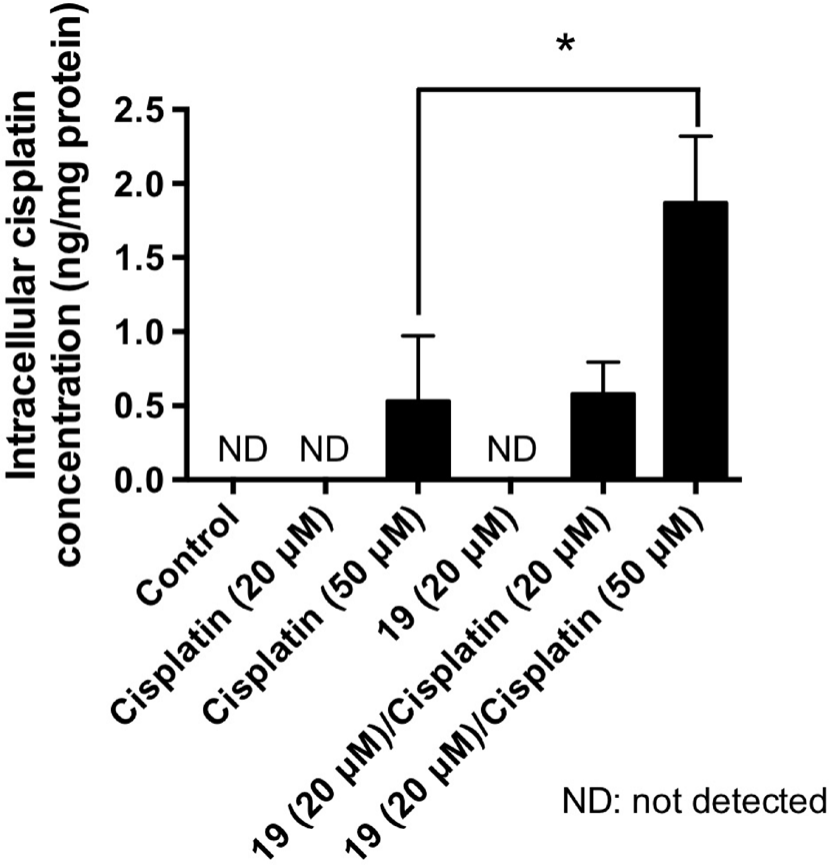
Compound 19 enhances the accumulation of intracellular cisplatin in ovarian CSC-LCs A2780cis ovarian cancer cells were stained with an ALDEFLUOR kit, and the cells with high ALDH activity were collected by cell sorting through flow cytometry. ALDH-high CSC-LCs were then treated as indicated for 3 h. The intracellular concentration of cisplatin was determined by flameless atomic absorption spectrometry (AAS). Data represent mean ± SD from three independent experiments. ^*^P < 0.05. ND, not detected.

Reduced intracellular accumulation of chemotherapeutic drugs is one of the major mechanisms underlying the drug resistance in ovarian cancer [21–23]. We have shown previously that thioxodihydroquinazolinone compounds enhance cisplatin-induced DNA damage response and apoptosis [17, 18]. We hypothesized that such enhanced cisplatin toxicity is partly contributed through increased intracellular platinum drug accumulation as a consequence of thioxodihydroquinazolinone exposure in cells. We sought to determine if compound 19 affects the intracellular accumulation of cisplatin in ovarian CSC-LCs. ALDH-high A2780cis CSC-LCs were sorted and collected by flow cytometry. CSC-LCs were then treated with agents alone or the combination immediately after isolation, because the isolated ovarian CSC-LCs population has been reported as unstable and quickly transitions back to a mixed population of CSC-LCs and non-CSC-LCs [24]. Three hours following treatment, CSC-LCs were lysed and the amount of intracellular cisplatin was determined by flameless atomic absorption spectrometry (AAS). As shown in Figure 3, intracellular cisplatin was not detectable after CSC-LCs were treated with cisplatin alone at 20 μM, which may underlie the platinum resistance of A2780cis CSC-LCs. In contrast, following the combination treatment of cisplatin (20 μM) with compound 19 (20 μM), the level of intracellular cisplatin was dramatically increased and was comparable to that when cells were treated with cisplatin alone at 50 μM. Compound 19 further enhanced the accumulation of intracellular cisplatin when cells were treated with the combination of compound 19 with 50 μM of cisplatin. The ability of compound 19 in facilitating the intracellular accumulation of cisplatin is therefore likely a critical mechanism underlying the enhanced cytotoxicity.

### The combination of compound 19 with cisplatin reduces sphere formation of cisplatin-resistant ovarian cancer cells

**Figure 4:**
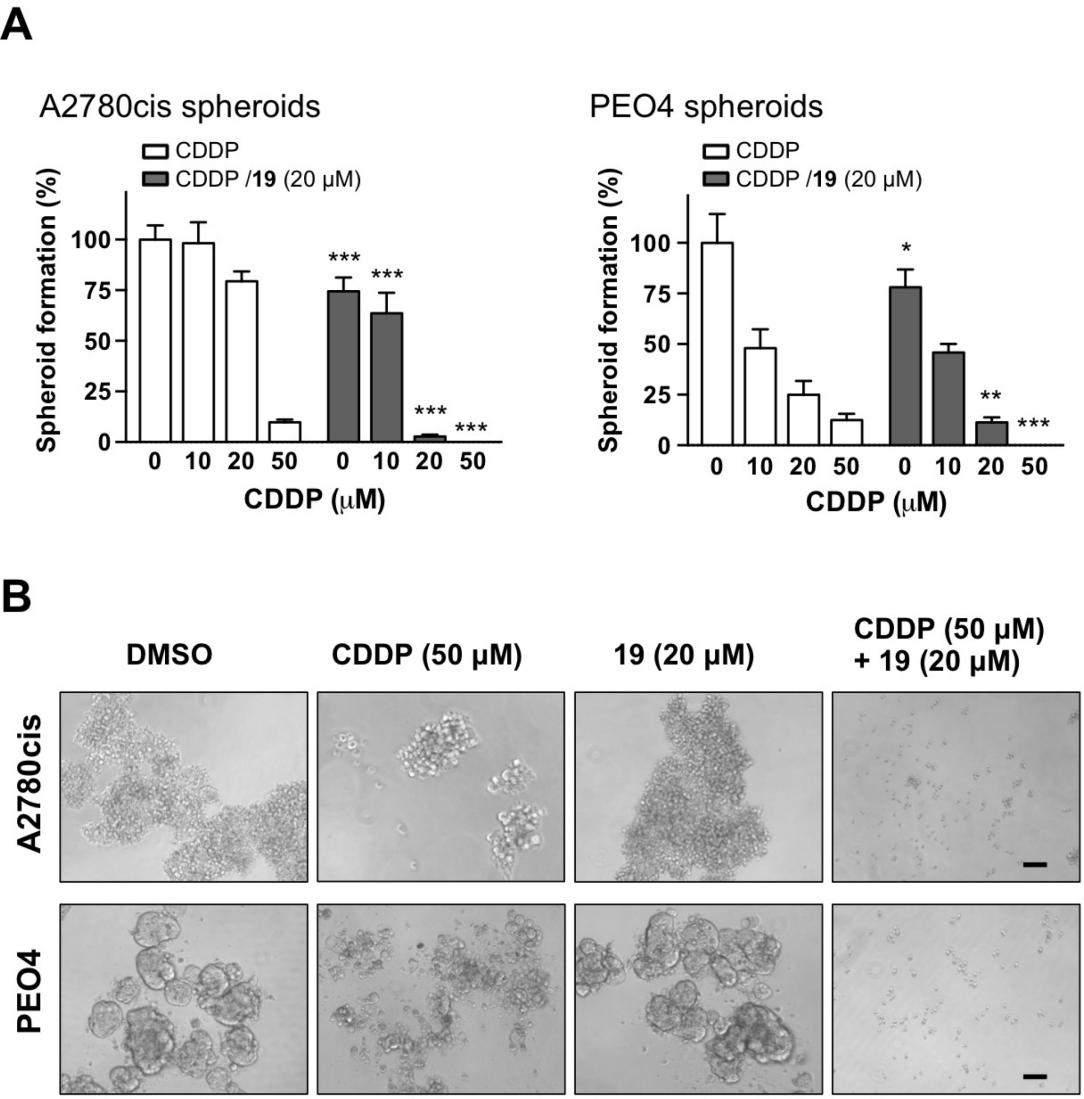
The combination of compound 19 with cisplatin prevents sphere formation from cisplatin-resistant cell subpopulation Monolayer A2780cis and PEO4 ovarian cancer cells were treated with cisplatin alone, compound 19 alone, or the combination for 3 h. Compounds were then washed away and the cells were transferred to ultra-low attachment tissue culture plates to allow the formation of spheroids from surviving population of cisplatin-resistant cells. After seven days, the growth of spheroids were quantified by MTS assay (**A**). Data represent mean ± SD. Student’s *t*-test was performed for comparisons between groups treated with the combination of cisplatin with compound 19 and groups treated with cisplatin alone at the same concentration. ^*^P < 0.05, ^**^P < 0.01, ^***^P < 0.001. (**B**) Representative images of spheroid formation at the indicated treatment conditions. Scale bars, 30 μm.

The recurrence of ovarian cancer can be attributed to the persistence of platinum-resistant CSC-LCs after initial chemotherapy. Ovarian CSC-LCs can be selected in cell culture by treatment with chemotherapeutic agents, and the surviving chemo resistant CSC-LCs can then be further enriched in spheroids [25]. Indeed, spheroid cells are found enriched for cells with stem cell-like properties [26], and cisplatin treatment leads to an increase in ALDH-high cell population [20]. A cisplatin-resistant spheroid model is thus more clinically relevant in ovarian cancer [27]. In order to investigate whether the combination is effective in eradicating drug-resistant cells with sphere-forming capability, we treated cisplatin-resistant A2780cis and PEO4 high-grade ovarian cancer cells in monolayer with cisplatin alone, compound 19 alone, or their combination for 3 hours. Such treatment can lead to the elimination of the drug-sensitive cell population, resulting in the selection of drug-resistant cells that can repopulate to form spheroids. It is also worth noting that PEO4 cells are resistant to multiple drugs, including PARP inhibitors, cisplatin, and 5-FU [28, 29]. In order to allow spheroid formation, 3 hours after treatment equal number of cells from each treatment group were transferred to drug-free media for seven days under stem cell-selective culture conditions, which consists of ultra-low attachment tissue culture plates and serum-free media containing growth factors EGF, bFGF, and N2 supplement-A. As shown in Figure 4, the combination of compound 19 and cisplatin significantly and dose-dependently reduced the formation of spheroids as compared to drug alone. For example, the combination of 20 μM cisplatin and 20 μM compound 19 reduced the formation and growth of A2780cis spheroids nearly 30-fold, when compared to cisplatin-alone treatment. This result indicates that the combination is more effective in reducing the sphere-forming ability of platinum-resistant cells following chemotherapy, which may therefore help prevent the recurrence of ovarian cancer.

### The combination of compound 19 with cisplatin prevents ovarian cancer metastasis in an *in vitro* model

**Figure 5:**
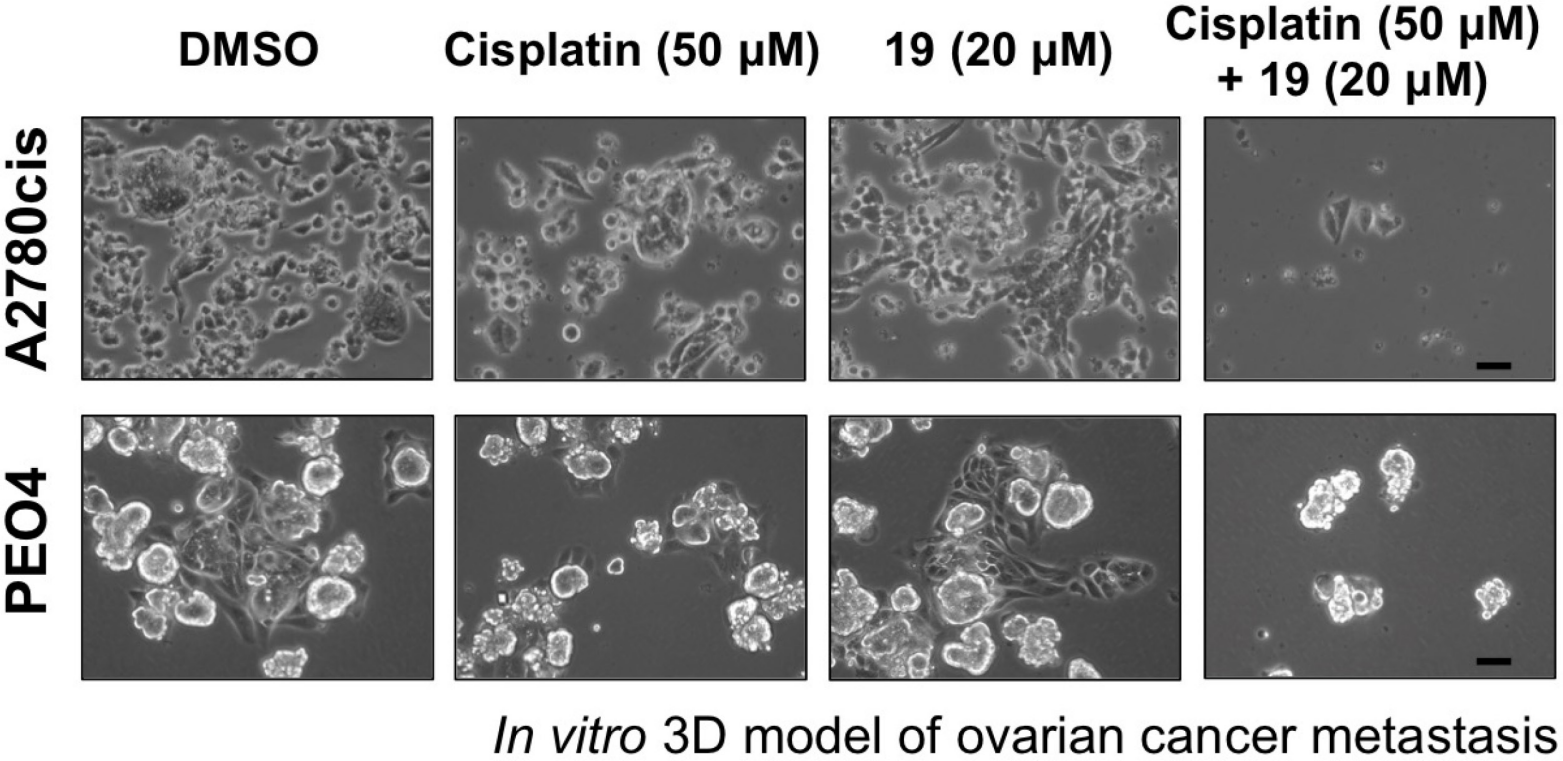
Exposure of ovarian cancer spheroids to the combination of compound 19 and cisplatin prevents spheroids reattachment and spreading Cisplatin-resistant A2780cis and PEO4 ovarian cancer cells were cultured in ultra-low attachment tissue culture plates for five days to allow spheroid formation. After 3 h treatment with compounds alone or the combination as indicated, spheroids were transferred to conventional tissue culture plastic to allow adhesion, growth, and spread of cells from spheroids in drug-free media. Images were taken 48 h after transferring spheroids to conventional tissue culture plate. Representative images are shown. Scale bars, 20 μm.

Metastasis is a major cause of clinical complication in ovarian cancer. The dissemination of ovarian cancer cells occurs directly in patient peritoneal cavity, which is a result of the migration of cancer cells from primary tumor sites into peritoneal fluids or ascites followed by implantation of those ascites cancer cells onto the mesothelial lining and further into tissues such as the omentum. Approximately 40% of ovarian cancer patients develop such metastatic ascites [30]. The malignant ovarian cancer cells present in ascites often aggregate to form spheroids [31, 32], which readily disaggregate upon adherence to mesothelial monolayers and have been shown enriched in cells with stem cell-like properties, including high ALDH activity [33]. We investigated whether the combination of compound 19 with cisplatin is able to reduce the metastatic potential of spheroids using an *in vitro* metastasis model, which is based on the ability of cultured spheroids to attach to the substratum and then disperse [34]. A2780cis and PEO4 ovarian cancer cells were cultured in ultra-low attachment tissue culture plates with stem cell-selective media for five days to allow the formation of spheroids. Spheroids were then treated with cisplatin alone, compound 19 alone, or the combination for 3 hours. After washing away the compounds, spheroids were transferred to conventional tissue culture plastic to allow adhesion and spreading of spheroid cells for 48 h. As shown in Figure 5, both cisplatin alone and compound 19 alone had only slight effects, whereas the combination dramatically reduced the attachment and subsequent spreading of spheroid cells.

### Compound 19 enhances cisplatin-induced apoptosis in ovarian cancer spheroids

**Figure 6:**
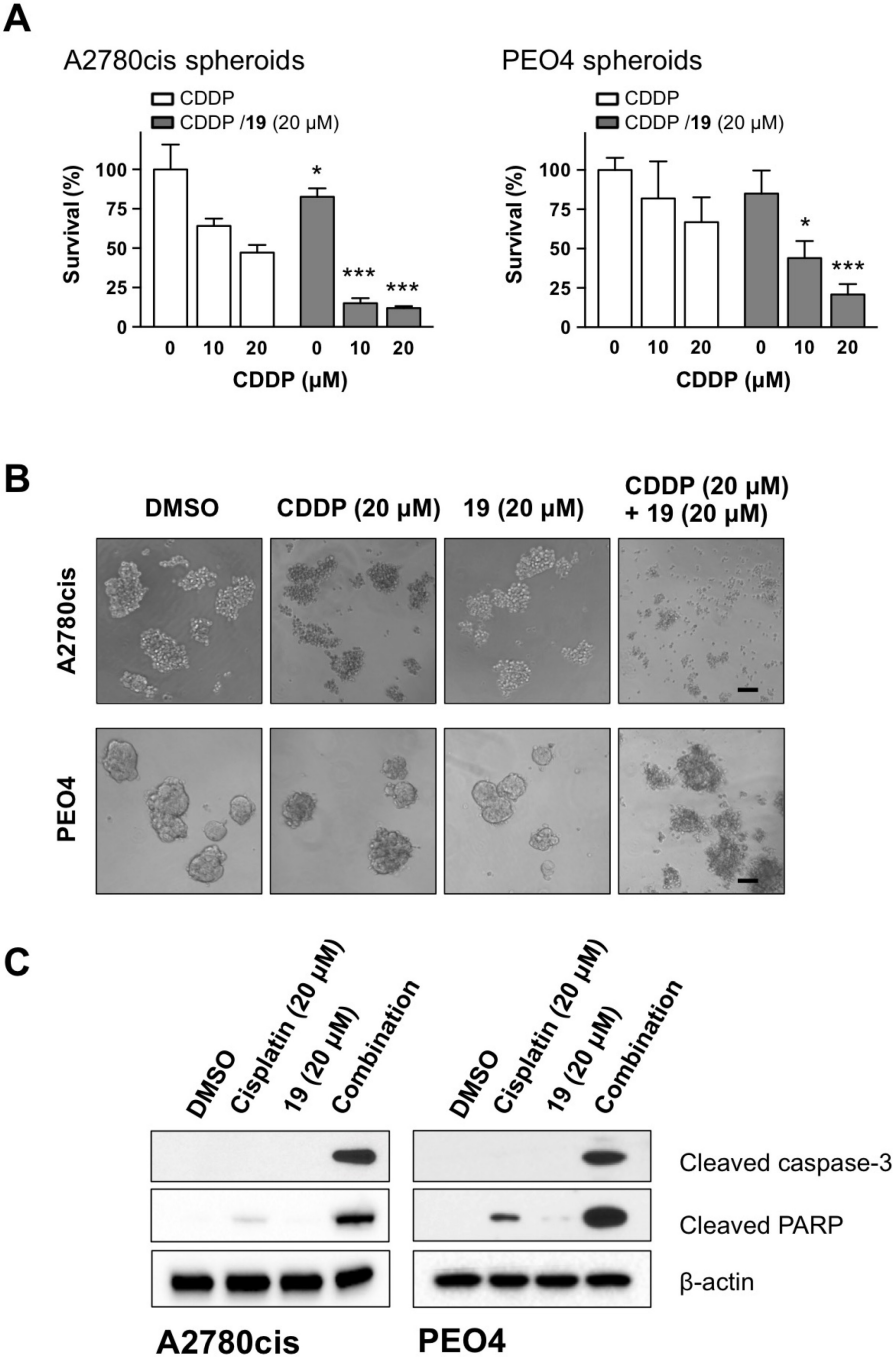
The combination of compound 19 with cisplatin enhances apoptosis in ovarian cancer spheroids (**A**) Five-day old spheroids generated from A2780cis and PEO4 ovarian cancer cells were treated as indicated for 48 h. Cell viability was determined by a MTS assay. Data represent mean ± SD. Student’s *t*-test was performed for comparisons between groups treated with the combination of cisplatin with compound 19 and groups treated with cisplatin alone at the same concentration. ^*^P < 0.05, ^**^P < 0.01, ^***^P < 0.001. (**B**) Representative images of spheroids 48 h after treatment with cisplatin alone, compound 19 alone, or the combination. Scale bars, 30 μm. (**C**) Apoptotic cell death of spheroids from A2780cis and PEO4 cells was evaluated by Western blot.

Tumor spheroids formed from drug-resistant malignant cells within patient ascites represent a major challenge to the efficacious treatment. Those spheroids possess unique survival mechanism to facilitate metastasis and recurrence. The formation of spheroids impedes the treatment efficacy by protecting cells from anoikis and reducing the penetration of chemotherapy drugs to internal cells of the spheroids [35, 36]. We therefore studied if the combination has direct effect on the survival of ovarian cancer spheroids formed from cisplatin-resistant cells. As shown in Figure 6A, the combination significantly reduced the viability of spheroids from both A2780cis and PEO4 cells in a dose-dependent manner, as compared to the treatment with cisplatin alone. Figure 6B shows that the combination is also effective in disrupting sphere structure, causing the dissociation of individual cancer cells from spheroids. We further observed a robust cleavage of caspase-3 and PARP after spheroids were treated with the combination of cisplatin and compound 19, as compared to agent-alone treatment (Figure 6C), indicating a stronger proapoptotic effect of the combination on tumor spheroids.

### Compound 19 enhances *in vivo* efficacy of cisplatin in platinum-resistant ovarian cancer xenografts

**Figure 7:**
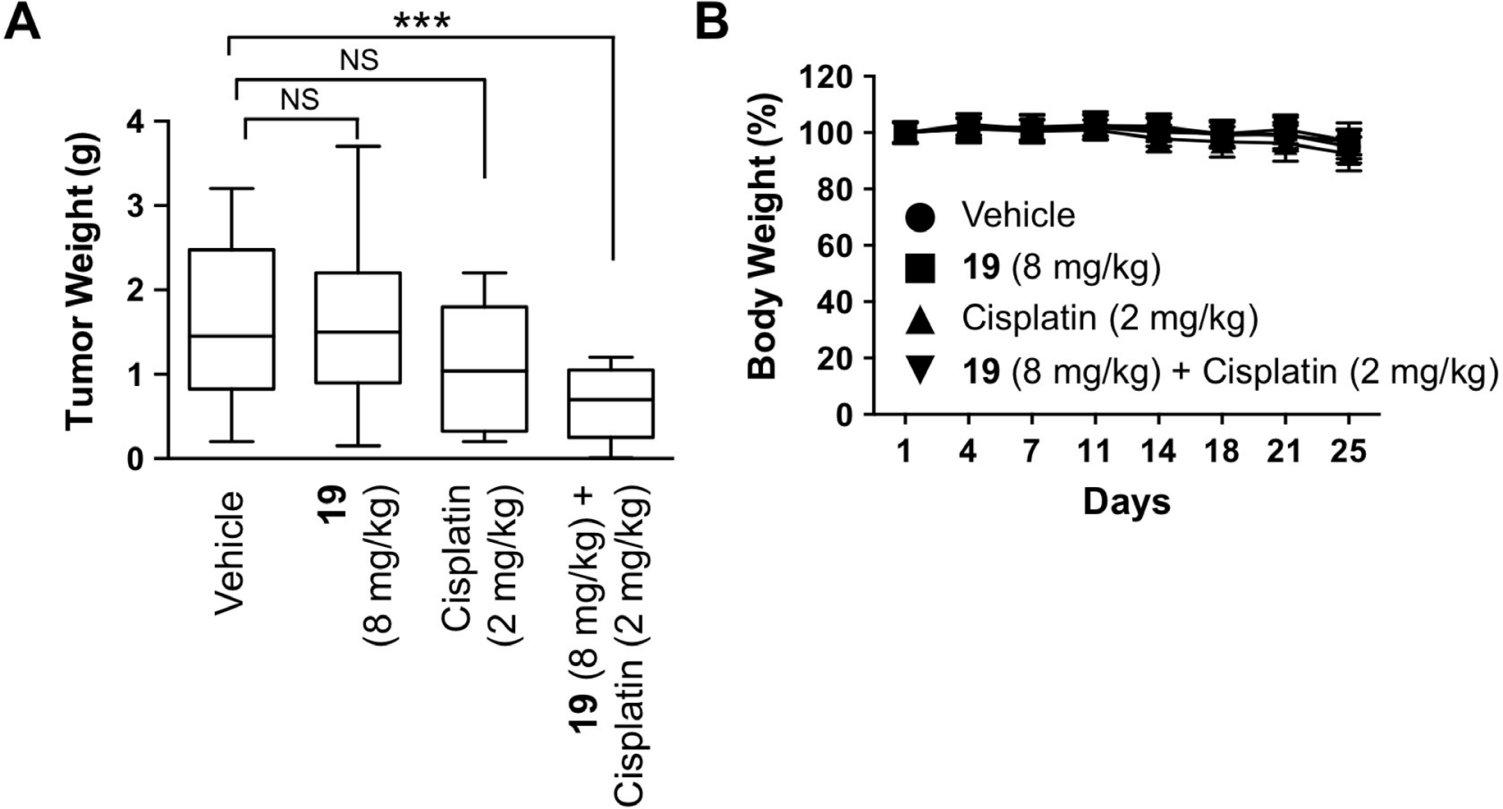
The combination of compound 19 with cisplatin reduces tumorigenesis *in vivo* A2780cis intraperitoneal ovarian cancer mouse model was utilized to study the effect of compound 19 alone, cisplatin alone, or the combination on the growth of ovarian tumor *in vivo*. 10 million A2780cis cells were injected intraperitoneally on day 1 in each Fox Chase SCID mouse. Beginning three days after tumor cell inoculation, drugs were administered intraperitoneally twice a week for three weeks. Body weight was measured twice a week. Mice were sacrificed on day 25, and intraperitoneally developed tumor nodules were collected and weighed. Each group contained 20 mice. Significance was determined by one-way ANOVA and Tukey multiple comparison test. ^***^P < 0.001, NS, non-significant.

To investigate the *in vivo* efficacy of compound 19, we studied the therapeutic potential of compound 19 alone and its combination with cisplatin using a metastatic intraperitoneal xenograft mouse model of human ovarian cancer established from cisplatin-resistant A2780cis cells. Three days after intraperitoneal A2780cis cell inoculation, compounds were administered simultaneously by intraperitoneal injections, twice a week for three weeks. Both the intraperitoneal inoculation of tumor cells and intraperitoneal injection of therapeutic drugs are considered to better recapitulate the native settings of ovarian cancer progression and have higher clinical relevance [37]. As shown in Figure 7, only the combination treatment with compound 19 (8 mg/kg) and cisplatin (2 mg/kg) was able to significantly reduce the weight of tumor nodules formed in mouse peritoneal cavity, while cisplatin alone did not have significant effect on tumor burden.

## DISCUSSION

Cancer stem cell-like cells (CSC-LCs) are sufficient to generate recurrent disease [38]. However, the therapeutic approaches that are able to eradicate CSC-LCs are limited. Our study demonstrated that the thioxodihydroquinazolinone compound 19 is a lead compound and represents a new class of agents that in combination with cisplatin are capable of eliminating cancer cells with stem cell characteristics in ovarian cancer. The combination of compound 19 with cisplatin diminished the cancer cell subpopulation with high ALDH activity, reduced sphere-forming ability of platinum-resistant cells, and suppressed spheroids-mediated metastasis. Importantly, the *in vivo* efficacy of compound 19 in combination with cisplatin in reducing tumor burden derived from cisplatin-resistant ovarian cancer cells further emphasized the preclinical and clinical potential of the combination strategy.

It has been shown that sub populations of chemotherapy-resistant cancer cells share certain characteristics with cancer stem cells [9]. Cancer stem cell-like cells are responsible for the relapse of ovarian cancer after chemotherapy due to their increased drug resistance. The expression and activity of ALDH have been used to define ovarian cancer cells with stem cell-like properties, including expressing transcription factors restricted in stem cells, forming tumor spheres, and high efficiently generating tumors *in vivo* [9, 39]. High ALDH activity has also been shown to overlap with other markers of ovarian CSC-LCs such as CD44 [40]. In both established cancer cell lines and primary ovarian tumors, cells with high ALDH activity are more resistant to platinum drugs [9]. High expression of ALDH is associated with poor prognosis of ovarian cancer patients [41]. Additionally, siRNA-mediated suppression of ALDH sensitized xenograft tumors to platinum and taxane treatment [42]. Cisplatin-resistant A2780cis ovarian cancer cells were derived from cisplatin-sensitive A2780 parental cells, and are about 10-fold more resistant to cisplatin than their parental A2780 cells [18]. An increased cell subpopulation with high ALDH activity has been observed in platinum- and taxane-resistant A2780 derivative cells [43, 44]. Furthermore, ALDH has been shown required to maintain stem cell-like characteristics [44]. Our data showed that the cell population with high ALDH activity is around 8% in A2780cis cells, and around 5% in primary ovarian cancer cells from ascites of a cisplatin-resistant end-stage ovarian cancer patient. The combination of compound 19 and cisplatin effectively diminished such CSC-LCs population whereas cisplatin alone was not able to achieve. By eliminating CSC-LCs, our combination strategy using compound 19 and cisplatin may help overcome drug resistance and reduce tumor recurrence, leading to improved treatment outcome.

The mechanism of drug resistance in CSC-LCs is extremely complex and remains elusive. One of the important mechanisms that differentiate CSC-LCs and non-CSC-LCs involves the expression of drug efflux pumps. High expression of drug efflux pumps in CSC-LCs leads to reduced intracellular drug effects and escape of cell death [21, 22]. Representative pumps include the major drug efflux ATP-binding cassette (ABC) transporters, breast cancer resistance protein (ABCG2), P-glycoprotein (ABCB1), and multidrug resistance-associated protein 2 (ABCC2). The high expression of drug efflux transporters is also the basis for a widely used method in identifying cancer stem cell-like cells through side populations that are capable of excluding Hoechst 33342 dye [45]. Ascites from recurrent ovarian cancer patients following chemotherapy have been found to contain a higher percentage of side populations that overexpress ABCB1 as compared to that of chemo-naive patients [46], indicating that drug efflux system is enhanced in drug-resistant cells with stem cell characteristics. Preventing drug efflux may therefore be an effective strategy to reverse drug resistance. It has been shown that suppression of ABCC2 by siRNA-mediated knockdown was able to restore sensitivity of cisplatin-resistant ovarian cancer cells to platinum and taxane treatment [47]. In addition to platinum efflux system, reduced platinum influx has also been observed in platinum-resistant ovarian cancer cells [48], and enhancing platinum influx by modulating copper transporter 2 (CTR2) was able to increase platinum toxicity [49]. Our previous work has shown that thioxodihydroquinazolinone compounds enhance cisplatin-induced DNA replication stress and DNA damage response signaling [17, 18]. In this study, we revealed that compound 19 is able to enhance the intracellular accumulation of cisplatin, which is conceivably a critical mechanism underlying the extensive DNA damage and cell death induced by the combination. Increased platinum drug retention in ovarian CSC-LCs could be a consequence of either reduced cisplatin efflux or enhanced influx or the combination of effects on both efflux and influx by the co-treatment with compound 19 and cisplatin. Further studies on the mechanism of action of compound 19 in influencing drug transport and the down-stream signaling including DNA damage response in CSC-LCs are warranted.

In addition to drug efflux pumps, mitochondria are another important player in the functions of stem cells and also cancer stem cells. Due to the critical role of mitochondria in cell death, targeting mitochondria in cancer stem cells may prove to be an effective strategy, regardless of the primary metabolic phenotypes of stem cells, glycolytic or oxidative [50–52]. Ovarian CSC-LCs have been reported to depend on OXPHOS [53] and ovarian cancer as a whole appears to rely on fatty acid beta-oxidation [54]. Niclosamide, through disrupting bioenergetics and redox regulation systems, has been shown to inhibit the growth of ovarian CSC-LCs [55]. We have shown previously that the combination of thioxodihydroquinazolinone analogs with cisplatin induces severe mitochondrial uncoupling and Bax/Bak-independent mitochondrial apoptosis in cisplatin-resistant ovarian cancer cells [17, 18]. Taken together, the strong effect of the combination of compound 19 and cisplatin on CSC-LCs may be a combined effect of enhanced intracellular accumulation of drugs and its detrimental effect on mitochondrial function.

CSC-LCs grow as spheroids in an anchorage-independent manner. Spheroids are commonly found in patient ascites, and are highly invasive into mesothelial surfaces to form secondary nodules [32]. It is well accepted that the formation of such spheroids in the peritoneal cavity of ovarian cancer patients contributes to drug resistance and recurrence. The three-dimensional (3D) culture of multicellular aggregates is a more relevant model of human tumors than conventional two-dimensional (2D) cell cultures on plastic. 3D spheroids are more resistant to chemotherapeutic drugs when compared to 2D monolayer cell cultures. Spheroid cells are also more aggressive in growth, invasion, and clonogenesis [26]. The formation of spheroids may thus constitute a key mechanism of ovarian tumor recurrence [56]. Spheroid formation is also a validated and widely employed approach for CSC enrichment, which selects for dominant self-renewal of CSC population [26, 57]. The analysis on spheroids genomic signature has revealed upregulated markers that are characteristics of CSCs, including ALDH, β-catenin, and c-KIT [33]. Due to the invasive behavior of spheroids, preventing the formation of the spheroids may represent an effective strategy for ovarian cancer management [58]. The effect of the combination of compound 19 and cisplatin in reducing the formation of spheroids indicates that the combination may be able to decrease stemness capability of CSC-LCs such as self-renewal or clonogenicity.

Collectively, our study suggests that the combination strategy consisting of thioxodihydroquinazolinone compounds and platinum drugs exerts a profound antitumor activity by effectively eliminating both CSC-LCs and non-CSC-LCs (the bulk of tumor cells). Future studies are needed to identify the critical factors or pathways that are responsible for increased intracellular accumulation of platinum drugs by thioxodihydroquinazolinones, the effect on stem cell metabolism and self-renewal, and the signaling pathways that lead to the cell death of CSC-LCs. It is conceivable that multiple targets are modified by the thioxodihydroquinazolinone compounds in combination with platinum drugs, leading to simultaneous disruption of key mechanisms of drug resistance. Understanding the mechanism of action of thioxodihydroquinazolinones may also aid in rational design and development of effective CSC-LCs-targeted therapy.

## MATERIALS AND METHODS

### Cell culture

The cisplatin-resistant human ovarian carcinoma cell line A2780cis was obtained from Sigma-Aldrich (St. Louis, MO). Ovarian carcinoma cell line PEO4 was kindly provided by Dr. Karyn J. Hansen (Magee-Womens Hospital of UPMC). Ovarian cancer patient ascites were obtained under an IRB protocol, Heath Sciences Tissue Bank IRB 0506140, approved by the University of Pittsburgh Cancer Institute. Primary epithelial ovarian cancer (EOC) cells presented in those ascites were isolated and cultured as described previously [59]. Cells were cultured in RPMI or DMEM media supplemented with 10% heat-inactivated fetal calf serum and 1% penicillin-streptomycin in 5% CO_2_ at 37°C. Spheroid cells were generated by culturing cells in serum-free DMEM/F12 media supplemented with 5 μg/mL heparin, 20 ng/mL human recombinant epidermal growth factor (EGF) (Thermo Fisher Scientific, Waltham, MA), 20 ng/mL basic fibroblast growth factor (bFGF) (Thermo Fisher Scientific, Waltham, MA), 1% N2 supplement-A (Stemcell Technologies Inc, Vancouver, BC, Canada), 1% glutamax, and 1% penicillin/streptomycin, in UltraLow Attachment plates (Corning Inc., Corning, NY).

### Reagents

Compound 19 (3-(2-ethylphenyl)-2-mercaptoquinazolin-4(3H)-one, PubChem CID: 2731304) was obtained from Enamine Ltd (Kiev, Ukraine). Other chemicals, unless specified, were from Sigma-Aldrich.

### ALDH activity assay

ALDH1 enzymatic activity was measured using the ALDEFLUOR™ assay kit from STEMCELL Technologies (Vancouver, BC, Canada). Briefly, cells were suspended in ALDEFLUOR assay buffer at a concentration of 2 × 10^5^ cells/mL, and stained with ALDEFLUOR reagent BODIPY-aminoacetaldehyde (BAAA), a fluorescent substrate for ALDH, at 37°C for 30 min. Cells were then resuspended in 200 μL of ice cold assay buffer containing 1 μg/mL propidium iodide (PI), and subjected to FACS analysis. Cells treated with ALDH inhibitor diethylaminobenzaldehyde (DEAB) served as negative control.

### Intracellular accumulation of cisplatin

ALDH-high A2780cis cells were collected by flow cytometry, and then exposed to various doses of cisplatin (0, 20, 50 μM) with or without the presence of 20 μM of compound 19 for 3 h. Cells were then washed to remove free drug and pelleted by centrifugation. To measure total intracellular platinum accumulation, cells were lysed with 0.25% triton X-100 and then sonicated. Platinum content in cell lysate was determined by flameless atomic absorption spectrometry (AAS) (Perkin-Elmer, Foster City, CA). Intracellular platinum levels were normalized by protein content and expressed as ng of platinum per mg of protein.

### *In vitro* 3D model of ovarian cancer metastasis

1 × 10^5^ cells per well of A2780cis and PEO4 ovarian cancer cells were cultured in 6-well ultra-low attachment tissue culture plates for five days to allow spheroids formation. Spheroids were then treated with compounds alone or combination for 3 h. After compounds were washed away, spheroids were transferred to conventional 6-well tissue culture plastic in drug-free media for 48 h to allow adhesion, growth, and spread of spheroid cells. After washing out unattached spheroids, phase contrast images of attached spheroid cells were captured using a Nikon Eclipse TS100 inverted microscope.

### Spheroids viability assay

A2780cis and PEO4 cells were cultured in 96-well ultra-low attachment tissue culture plates for five days to allow spheroids formation. Spheroids were then treated with compounds alone or combination for 48 h. The survival of spheroids was determined using the CellTiter 96^®^ AQueous One Solution Cell Proliferation Assay kit (Promega, Madison, WI), according to the manufacturer’s instructions.

### Western blot analysis

Western blot was performed as we previously described [60]. Primary antibodies against cleaved Caspase-3 and cleaved PARP were obtained from Cell Signaling Technology (Beverly, MA), and antibody against β-actin was obtained from Sigma-Aldrich (St. Louis, MO).

### *In vivo* xenograft studies

Animal studies were approved by the Institutional Animal Use and Care Committee (IACUC) of the University of Pittsburgh (Protocol 15043367). Female CB17SCID mice (6–8 weeks) were purchased from Charles River (Wilmington, MA). Mice were housed five per cage at Hillman Cancer Center animal facility and maintained according to University of Pittsburgh IACUC-directed laboratory conditions. Veterinary care was provided by the Division of Laboratory Animal Research of the University of Pittsburgh. Each mouse was inoculated intraperitoneally with 10 million A2780cis cells suspended in 500 μL PBS. Beginning three days after inoculation of A2780cis cells, mice were treated intraperitoneally with cisplatin (2 mg/kg) alone, compound 19 (8 mg/kg) alone, or the combination of cisplatin (2 mg/kg) and compound 19 (8 mg/kg), twice a week for 3 weeks. Cisplatin was dissolved in saline, and compound 19 was prepared in a formulation containing solutol HS15/ DMA/ ethanol/ saline at a ratio of 30:10:15:45 (%volume). Animals were observed daily, and any signs of distress were reported to the veterinary staff for evaluation. Body weight was monitored twice a week. To determine treatment efficacy, mice were sacrificed and tumor nodules inside peritoneal cavity were collected and weighed.

### Statistical analysis

Data were expressed as mean ± standard deviation. A Student’s *t*-test was used for the comparisons between two groups. ANOVA was used to make comparisons between multiple groups. *P* < 0.05 was considered statistically significant.
